# An Analysis Review of Detection Coronavirus Disease 2019 (COVID-19) Based on Biosensor Application

**DOI:** 10.3390/s20236764

**Published:** 2020-11-26

**Authors:** Bakr Ahmed Taha, Yousif Al Mashhadany, Mohd Hadri Hafiz Mokhtar, Mohd Saiful Dzulkefly Bin Zan, Norhana Arsad

**Affiliations:** 1 UKM—Department of Electrical, Electronic and Systems Engineering, Faculty of Engineering and Built Environment, Universiti Kebangsaan Malaysia, Bangi 43600, Malaysia; p103537@siswa.ukm.edu.my (B.A.T.); hadri@ukm.edu.my (M.H.H.M.); saifuldzul@ukm.edu.my (M.S.D.B.Z.); 2 Department of Electrical Engineering, College of Engineering, University of Anbar, Anbar 00964, Iraq; yousif.mohammed@uoanbar.edu.iq

**Keywords:** COVID-19 detection, biosensor application, COVID-19 transmission styles, sensors interaction, artificial intelligence

## Abstract

Timely detection and diagnosis are essentially needed to guide outbreak measures and infection control. It is vital to improve healthcare quality in public places, markets, schools and airports and provide useful insights into the technological environment and help researchers acknowledge the choices and gaps available in this field. In this narrative review, the detection of coronavirus disease 2019 (COVID-19) technologies is summarized and discussed with a comparison between them from several aspects to arrive at an accurate decision on the feasibility of applying the best of these techniques in the biosensors that operate using laser detection technology. The collection of data in this analysis was done by using six reliable academic databases, namely, Science Direct, IEEE Xplore, Scopus, Web of Science, Google Scholar and PubMed. This review includes an analysis review of three highlights: evaluating the hazard of pandemic COVID-19 transmission styles and comparing them with Severe Acute Respiratory Syndrome (SARS) and Middle East Respiratory Syndrome (MERS) to identify the main causes of the virus spreading, a critical analysis to diagnose coronavirus disease 2019 (COVID-19) based on artificial intelligence using CT scans and CXR images and types of biosensors. Finally, we select the best methods that can potentially stop the propagation of the coronavirus pandemic.

## 1. Introduction and Overview of Coronaviruses

There are, to date, 15,033,861 cases of Coronavirus disease (COVID-19) including 618,061 deaths worldwide. Due to the lockdown because of coronavirus, many of global activities have stopped, several businesses have reduced their operations and more people expect their jobs to be lost [1]. The following part describes the structure and genome of SARS-CoV-2.

### 1.1. SARS-CoV-2 Structure and Genome

**Figure 1:**
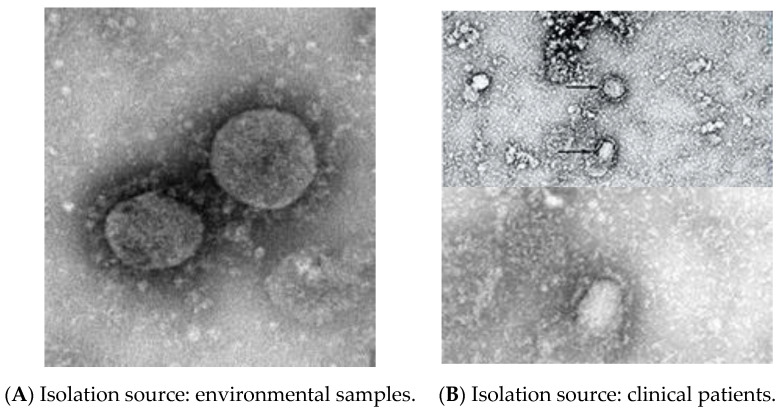
Taxonomy information of 2019-nCoV, β coronavirus (**A**). Environmental sample of 2019-nCoV, β coronavirus on 22 January 2020 at Wuhan, Hubei Province, China, (**B**). Clinical patient sample of 2019-nCoV, β coronavirus on 6 January 2020 at Wuhan, Hubei Province, China. Source: National Pathogen Resource Collection Center (National Institute for Viral Disease Control and Prevention under Chinese Center for Disease Control and Prevention). URL: http://nmdc.cn/nCov/en.

Coronaviruses (CoVs) are single-strand RNA viruses. CoVs are classified into four genotypes, alpha-coronavirus (αCoV), beta-coronavirus (βCoV), delta-coronavirus (δCoV) and gamma-coronavirus (γCoV). The category of viruses that are highly infectious in bats and rats are αCoV and βCoV, while δCoV and γCoV are present in birds [2,3]. Coronaviruses have been classified into Severe Acute Respiratory Syndrome CoV (SARS-CoV) in China in 2003, Middle East Respiratory Syndrome CoV (MERS-CoV) in Saudi Arabia in 2012 and Severe Acute Respiratory Syndrome CoV-2 (SARS-CoV-2) or Coronavirus Disease (COVID-19) in China in 2019. COVID-19 appears to only be slightly different from SARS-CoV in its clinical characteristics. The COVID-19 genome involves four main protein molecules: Spike (S), Membrane (M), Envelope (E) and the Nucleocapsid (N) protein [4]. SARS-COV-2 is spread even more widely in the community [5,6]. Coronaviruses, SARS-CoV-2, are a wrapped virus approximately 60–140 nm in diameter and are roughly spherical or mildly pleomorphic [7]. Figure 1 shows the taxonomy information of 2019-nCoV, β coronavirus for an environmental sample and clinical patient sample from Wuhan, Hubei Province, China. The following section describes/narrates the background of the virus detection.

### 1.2. Background Virus Detection

A nanofluidic channels technique with optical interferometry is used to detect, size and classify viruses and nanoparticles [8]. The research describes the process of designing a portable microscope based on a fluorescence platform fixed on a smartphone for imaging viruses and nanoparticles [9]. Surface plasmon resonance (SPR) techniques were used for processing inside optical sensors for chemical and biochemical applications such as viruses, DNA and bacteria as discussed in this article [10]. A surface plasmon resonance imaging (SPRI) technique was utilized to detect individual microparticles and nanoparticles in liquids such as viruses [11]. The development of biosensor device structures based on a photonic crystal fiber (PCF) to detect small sized molecules has been reported [12]. An image processing technique based on interferometry was employed to detect coronavirus [13]. An optical biosensor system for detecting influenza viruses was developed. The method used was a Mach–Zehnder waveguide to identify a virus exceeding 100 nm [14]. An optical biosensor based on an SPR technique was used with a coating layer from nanoparticle gold of 40 nm to detect the avian influenza virus [15]. A convolutional neural network (CNN) has been used to identify and classify viruses using SPR in an optical fiber [16]. It used nanographene and silver (Ag) material to coat an optical fiber for DNA sensing and environmental monitoring [17]. A study showed that implementing an optical biosensor for the multi-channel smartphone spectrometer (MASS) can be used to detect nanoparticles [18]. A Localized Surface Plasmon Resonance (LSPR) technique based on a gold nanoparticle (AuNP) modified to detect the influenza virus was reported [19]. Enhancement of the plasma assisted nano-object microscopy (PAMONO) sensor used a deep neural network (DNN) technique for the detection of nanoparticles at a low signal noise ratio (SNR) [20]. The PAMONO sensor technique was used with the connected SPR platform to detect viruses without supervision [21]. The biosensor LSPR device coupling SPR platform was improved by using a graphene oxide/silver coating to identify viruses [22]. A study showed the use of a nanolaser method in biological optical sensing [23]. A photonic crystal fiber biosensor based on a porous silicon structure presented was used to detect small chemical molecules [24]. A developed biosensor LSPR used a surface-enhanced Raman scattering (SERS) multiplex to detect MERS-CoV [25]. A SERS biosensor was developed based on lateral flow immunoassay (LFIA) to detect the influenza virus [26]. AI techniques have been used to diagnose and classify COVID-19 via x-ray and CT scan images [27]. A hybrid model (deep learning and machine learning) was presented to classify coronavirus disease images as COVID-19 or normal [28]. Coronavirus disease has been identified based on classifying x-ray images by using a CNN deep learning technique [29]. Image techniques with fluorescence have been used to improve single virus tracking [30]. A study showed that the computerized tomography (CT) technique was accurate for recognizing COVID-19 [31]. It used deep learning CNN tools to diagnose and classify coronavirus disease by using chest x-ray (CXR) images [32]. A decision-making aid to radiologists was developed to accelerate the diagnosis of COVID-19 by using deep learning CNN algorithms [33]. AI techniques have been used for SERS identification [34]. A study has been developed using a biosensor based on a field effect transistor (FET) method of detecting SARS-CoV-2 virus [35]. A hybrid generative adversarial network (GAN) with in-depth coronavirus detection learning using x-ray chest images has been reported [36]. Validation and classification of the COVID-19 virus in chest x-ray images used the deep learning CNN method [37]. The microfluidic chip technique by using AI to detect viruses has been improved [38]. It demonstrated optical biosensor LSPR for the possible detection of coronavirus disease [39]. The researchers presented an overview of optical biosensors that were used to detect the COVID-19 virus [40]. An optical biosensor device, a compact device based on the SPR with a gold nanoparticle coating, was developed for SARS-CoV-2 virus identification [41]. AI has been used to fight COVID-19 through tracking, diagnosis and social control [42]. AI profound learning techniques of COVID-19 have been presumed to be derived from individual image features [43]. One diagnostic method for COVID-19 used low-frequency Raman (LFR) spectroscopy [44]. A new approach is the dual-functional SPR, which combines the photo-thermal effect in a biosensor LSPR for SARS-CoV-2 virus detection [45]. Deep neural networks with multi-class x-ray COVID-19 images have been used to diagnose normal, pneumonia and COVID-19 cases [46]. Proposed profound learning algorithms have used CNN to classify SARS-CoV-2 by using images for chest image CT scans [47]. Deep learning methods have been used to help radiologists automatically diagnose positive or negative coronavirus disease cases [48]. Research has used immunosensors dependent on LPFG for the detection of viruses [49]. The next section discusses the COVID-19 styles of transmission.

## 2. Taxonomy of Literature Research of Coronavirus Diseases (COVID-19)

**Figure 2:**
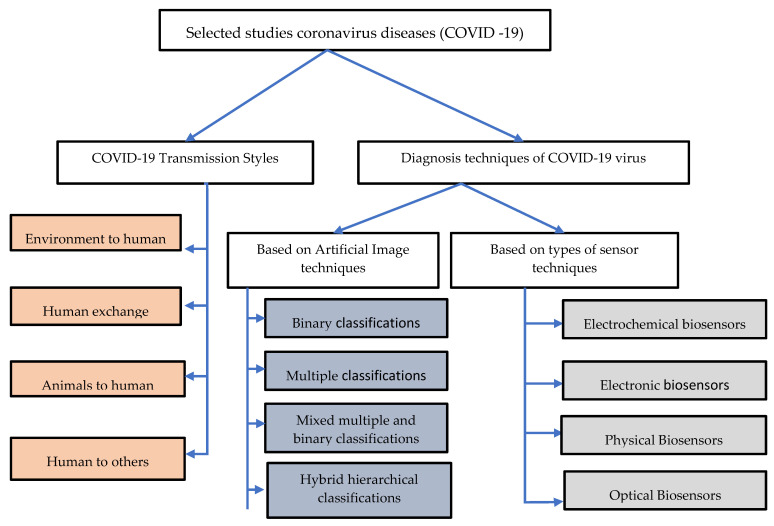
Taxonomy of literature research on coronavirus disease (COVID-19).

Our review identified, examined and analyzed 17 empirical studies of transmission styles of the COVID-19 virus and 24 empirical studies of the diagnostic techniques. Academic digital repositories were utilized for the extraction of relevant literature such as Science Direct (which offers various scientific research across all fields), Scopus (which provides ample work coverage from all disciplines), Web of Science (which shows extensive coverage of various subjects and researchers in all literature), IEEE (which is recognized as scientifically accurate and protected by the multidisciplinary information), PubMed (which also covers several topics including an interdisciplinary emphasis on research related to medicine and technology) and Google Scholar, as shown in Figure 2. The following section gives a taxonomy of literature research related to COVID-19.

## 3. COVID-19 Transmission Styles

This section summarizes and describes the potential styles of transmission for SARS-CoV-2. The standard mode of transmission of coronavirus disease we can classify in to four parts: environment to human, human exchange, animals to human and human to others. The next section discusses the process of coronavirus transmission.

### 3.1. Environment to Human

**Figure 3:**
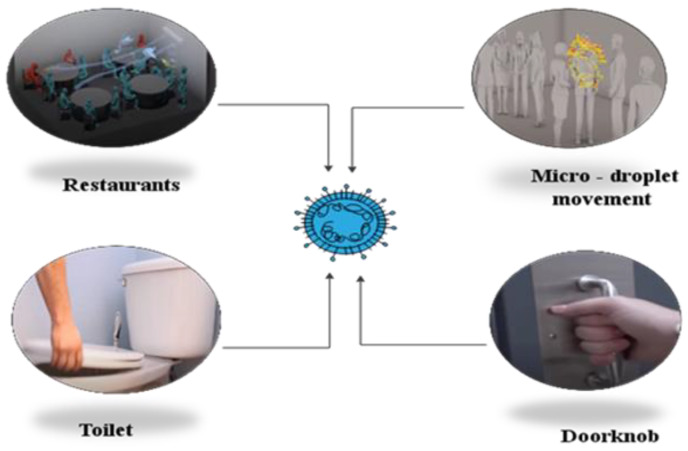
Transmission concepts for the COVID-19 virus from environment to human.

The built environment (BE) is a collection of places people have made including houses, vehicles, highways, public transport and other building spaces. Most citizens spend 90% of their daily lives in the BE [50]. Preliminary studies indicate that SARS-CoV-2 can probably continue to survive on a surface ranging from 2 h to 5 days. It was estimated that the virus can survive at a rate of 40% humidity for an extended period on plastic surfaces for approximately 72 h, stainless steel 48 h, cardboard 8 h, copper 4 h and aerosols 3 h [51]. Built environment surfaces are a possible factor for COVID-19 spread by causing close interactions between people. A dense population in buildings raises the degree of indoor activity due to business and communication via direct contact between individuals, enabling the accrual of microorganisms associated with humans and environmentally mediated contact with surfaces. Abiotic, air and surface pollution by SARS-CoV-2 in the hospital room has been detected [52,53]. Research has shown that the pandemic transmission of coronaviruses such as extreme SARS-CoV and MERS-CoV can live on surfaces for long period of time, sometimes for months [54]. Exploration microbiology ordered this in the built environment and essential data about the SARS-CoV-2 to give noteworthy and attainable direction to BE leaders, building administrators and every indoor administrator to decrease the transmission of highly contagious ailments through natural interceded pathways [55,56]. It presented an in-depth study of the detection of SARS-CoV-2 in a water environment [57]. A study showed a proven detection of the SARS-CoV-2 virus in hospital toilets and rooms [58,59]. Transmission concepts for the COVID-19 virus from environment to human are shown in Figure 3.

### 3.2. Human Exchange

**Figure 4:**
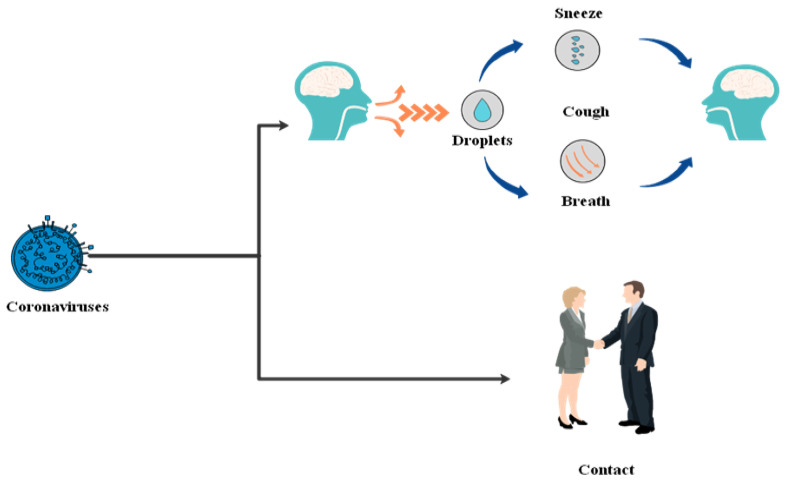
Transmission concepts for COVID-19 virus from human exchange.

Human coronaviruses are divided into alpha and beta. They represent families of enveloped, single-stranded RNA viruses with surface spike projections. The rapid appearance and transmission of a novel virus called β coronavirus resulted in the 2019 global coronavirus pandemic COVID-19 associated with colossal death [60,61]. A few studies have been issued to explain the pathophysiological aspects of the COVID-19 virus and propagation mechanism behaviors of the virus based on human exchange transmission [62]. Willcox et al. studied the coronavirus disease affected with conjunctival eye surface corneal, It showed that infection could lead to mild signs and pneumonia symptoms, which is rarely found [63,64]. Respiratory viruses are typically mostly symptomatic and mostly contagious. However, there is growing evidence that human exchange transmission during the asymptomatic incubation period of COVID-19 is estimated to be between 2–10 days [65]. The normal SARS-CoV-2 transmission systems include oral, nasal and eye mucus transmission and direct transmissions such as coughs and sneezes [66,67]. Transmission concepts for the COVID-19 virus from human exchange are shown below in Figure 4.

### 3.3. Animals to Human

**Figure 5:**
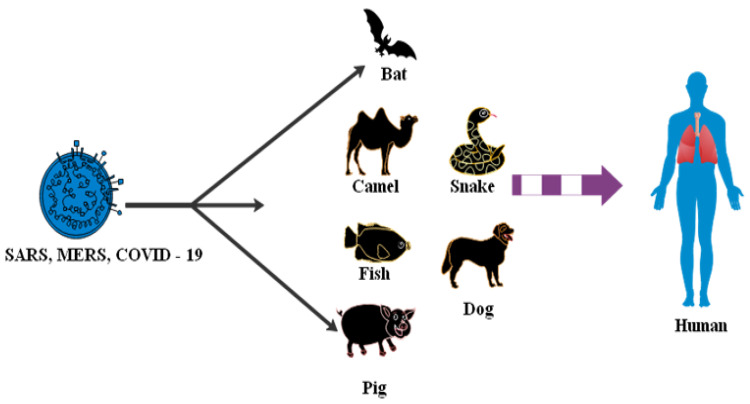
Transmission concepts for COVID-19 virus from animals to human.

Many items require a common health strategy to address and eliminate outbreaks of a related virus. Potentially, SARS-CoV-2 is transmitted to humans by spillover from bats or an unknown animal host. Studies have shown coronavirus transmitted to humans from a bat [68,69]. Bio-aerosol microscopic airborne particles pose widespread human and animal threats [70]. The study presented an analysis of the SARS-CoV-2 transmission theory from a list of animal types such as avian, swine, porcine, bovine, canine, seafood, frogs and camels sold on the market in Wuhan [71]. A total of 33 data samples was collected of SARS-CoV-2 from 585 environmental aspects in the seafood market [72]. A depth study of SARS-CoV, MERS-CoV and SARS-CoV-2 was related to different bat species [73,74]. Transmission concepts for COVID-19 virus from animals to human are shown below in Figure 5.

### 3.4. Human to Others

Other modes of transmission of SARS-CoV-2 RNA have further been detected in different biological samples including the feces and urine of some patients. However, there are no reported records of SARS-CoV-2 transfer by feces or urine [75]. An analysis of COVID-19’s genetic materials in sewage can alert against an epidemic. SARS-CoV-2 can be dealt with via sewage. The evolution of the SARS-CoV-2 virus in water, soils and other environmental compartments can be classified through sewage [51,76] A study presented a literature review on inanimate surfaces concerning the life cycle of human coronaviruses. The transmission of viruses transmitted via droplets, contaminated hands or surfaces were identified [77]. It showed SARS-CoV-2 detection in Australia’s untreated sewage [78,79]. There has been detection of the 2019 novel coronavirus (2019-nCoV) in hospital in infected patients’ rooms. The methodology was to understand the virus size distribution in the air and environmental contamination patterns were necessary for infection control policies [30,80].

#### Analysis Outcome of COVID-19 Transmission

**Figure 6:**
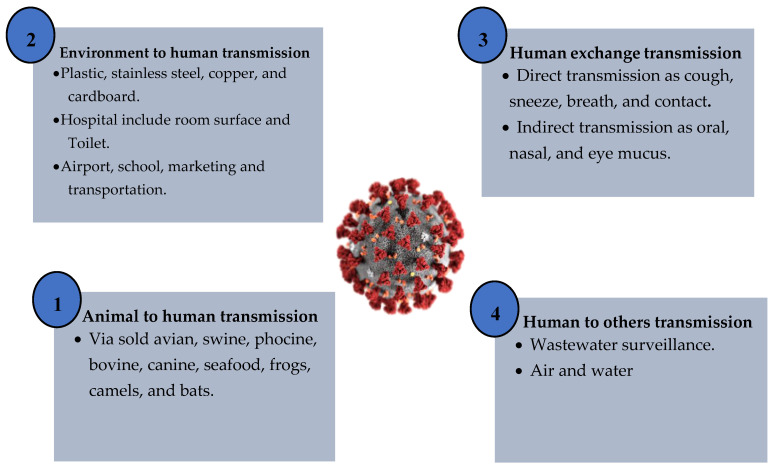
Categories of hypothesized SARS-CoV-2 virus origin and a common path of outbreak zoonotic coronavirus transmission.

An analysis of eight studies showed that SARS-CoV-2, SARS-CoV and MERS-CoV can continue on surfaces such as glass, metal, plastic, copper and cardboard for up to nine days. Table 1 shows an analysis of category styles of the transmission of coronavirus. Based on available information including previously mentioned reports and recommendations of the WHO, COVID-19 is now a global public health problem and worldwide mortality is rising rapidly [81,82,83]. It is essential to know the potential mechanisms of COVID-19 transmission and human behavior in addition to factors that probably support and decrease the spread of coronaviruses. Figure 6 shows the categories of hypothesized SARS-CoV-2 virus origin and a common path of outbreak zoonotic coronavirus transmission. The density of people in buildings raises the degree of indoor activity due to interaction and communication via direct contact between individuals, enabling the accrual of microorganisms associated with humans and environmentally mediated contact with abiotic surfaces. The spread effect of COVID-19 has been classified into four categories: extra strong, strong, middle and low. However, understanding the mechanism of transmission in the air and patterns of environmental pollution of SARS-CoV-2 is fundamental for infection prevention strategies. Respiratory secretions or droplets released through infected people may contaminate surfaces and objects for periods extending from hours to days based on the atmosphere including humidity, temperature and surface type. Therefore, an infection can usually occur indirectly through contacting surfaces in the live environment or things polluted with viruses from an infected person, resulting in touching the mouth, nose, or eyes [84,85].

## 4. Diagnosis Techniques of the COVID-19 Virus

This section analysis critical study reviews of the diagnosis methods of detection of COVID-19. These papers have been divided into various topics and techniques. Selected works were classified into broad categories based on artificial image techniques and types of sensor applications for fighting COVID-19.

### 4.1. Based on Artificial Intelligence Techniques

The taxonomy of AI research literature used to identify and recognize medical images of COVID-19 is based on four techniques: binary classifications, multiple classifications, mixed multiple class and binary classifications and hybrid multiple class and hierarchical classifications [86,87].

#### 4.1.1. Binary Classifications

The binary classifications, problems refer to classifying with only two different classes. The capacity of deep learning approaches to COVID-19 diagnosis based on medical images obtained from CT have been demonstrated. Regarding the class labels used to detect the presence of infections, this study focused on false-negative (FN) findings that endangered others and affected the decisions whether to continue the monitoring or discharge the patient. The dataset used consisted of ten patients’ details. In the RT-PCR test for COVID-19, two out of ten negative cases were reported as positive. In the previous version, RT-PCR was clearly shown and reported [88,89].

#### 4.1.2. Multiple Classifications

The multiple class grouping poses various problems and challenges. The creation of a COVID-19 severity scoring method was involved. It presented a COVID diagnosis net, a search for an AI method for coronavirus disease based on a deep squeeze net with optimization to detect COVID-19. The rate of detection of 98.3% for COVID-19, pneumonia and normal cases [90]. The study used a system to assign patients to severity-specific categories, which were extreme and moderate/mild according to the WHO classifications. Accordingly, 13,500 COVID-19 patients obtained the dataset used. An early review has shown that 93.6% of patients were appropriately established, 0.8% of the patients’ condition were underestimated and 5.7% were overestimated [91]. COVID-19 has been established using a deep learning method, a MobileNetV2 module and a squeeze net. Fuzzy color technology was used as a pre-processing step to restructure data classes and to combine organized images with original images. Efficient features with a total classification rate of 99.27% were grouped and categorized using support vector machines (SVMs) [92,93]. A neural network convolutional technique was introduced and a minimal number of parameters were employed in the technique to diagnose coronavirus via a statistical analysis of the possible chest x-ray imaging biomarkers [32].

#### 4.1.3. Mixed Multiple and Binary Classifications

This subcluster includes articles that focused on multiple integrated and binary classification problems. The use of AI to help the research of radiologists and classifications have been emphasized. We suggest that it is possible to control the progress of the disease by applying AI in COVID-19 infection [94]. In addition to multiclassification (COVID-19 vs. no findings. pneumonia), a model for the identification of COVID-19 was presented with 125 x-ray images for the accurate diagnosis of a binary classification (COVID-19 vs. normal). The model’s reliability for binary classes was 98.08% and for multiple class cases 87.02% [46]. Artificial intelligence techniques have been used for protecting health care workers and curbing the spread of COVID-19 [95].

#### 4.1.4. Hybrid Hierarchical

Another challenge for classes is the hierarchical grouping where the learning output is categorized by class taxonomy. The hierarchical classification is defined as follows: every class, which is divided into sub-classes or grouped into super-classes, is to be categorized into one and only one class. The hierarchy is established during classification and cannot be modified [86]. A classification approach was identified and developed for COVID-19 and pneumonia from different healthy lung types that took into account hierarchical and multiclass views [96]. The hybrid platform for COVID-19 detection used an improves marine predator algorithm (IMPA) and a ranking-based strategy for decreasing diversity in order to obtain particle numbers that cannot seek acceptable solutions for consecutive iterations. For IMPA accuracy testing, nine x-ray chest photos were used [97].

##### Analysis Outcome of Diagnostic COVID-19 Based on Artificial Intelligence Techniques

An analysis of ten studies showed AI techniques had been widely used for the classification of coronaviruses and healthcare [98]. It is one of the essential points that has been focused on in this review. The implementation of deep learning techniques and algorithms to identify a new coronavirus such as COVID-19 has many unique challenges. Although deep learning techniques are highly automated, a wide range of data is required to develop a robust diagnostic system. As COVID-19 is very new to research, the lack of useful data is a major diagnostic challenge. In a few cases, the imagery information available for COVID-19 patients is incomplete, noisy, unclear and inaccurate. Training a profound learning system with such large and varied datasets is very complicated. Many problems such as data consistency, non-linearity and missing values need to be addressed. Table 2 presents a comprehensive in-depth study of artificial intelligence technology (AI) employed for 2019 coronavirus classification and the identification of COVID-19 images.

### 4.2. Based on Biosensors Application

A biosensor is a device coupled with a transducer that produces a signal comparative and analysis detection of concentrations of biological elements such as nucleic acids, DNA, RNA, antibodies and cell receptors. According to studies that have been collected, 12 articles in this review paper are regarding the type of sensors used in the detection and diagnosis of COVID-19. This final set was divided into four groups, namely, optical biosensors, electronic biosensors, electrochemical biosensors and physical biosensors. This section discusses a biosensor taxonomy used to detect COVID-19 viruses in the literature. Typically, the biosensor system consisted of four main modules: a biosensor, a bioreceptor, a transducer and a digital output detector [99].

**Figure 7:**
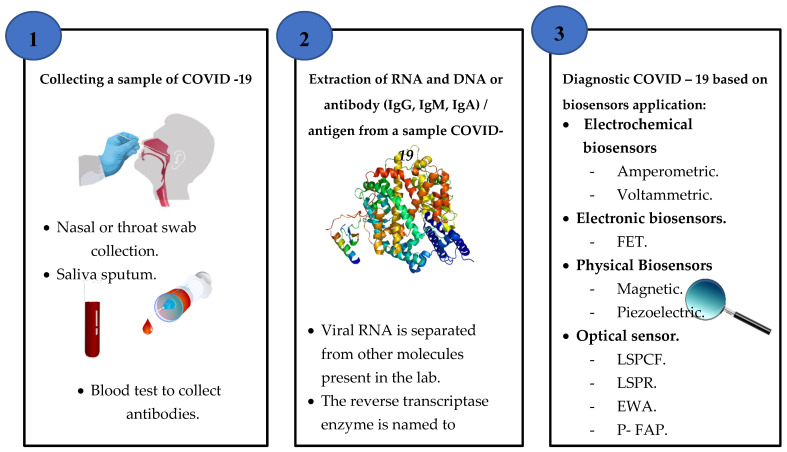
A schematic diagram of the collecting and extraction sample steps to detect COVID-19 based on biosensor applications.

Diagnosis methods of coronavirus disease (COVID-19) based on the application of biosensors are evaluated according to data extracted from literature studies. However, most of the new sensors in both research and marketing focus on electrical or optical receptors [100]. These include hand-held portable devices, ingestible sensors, screen-printed electrodes, wearable devices and single molecule sensors [101,102,103,104,105]. Collecting and extraction sample steps to detect COVID-19 based on biosensor application is shown in Figure 7.

#### 4.2.1. Electrochemical Biosensors

Electrochemical biosensors are biochemical concentrations of information transformation into an analytically useful signal via a current or voltage. This section includes two different publication works. An electrochemical biosensor device (eCovSens) was developed and compared with an active commercial tool to detect COVID-19 spike protein antigens in spittle samples. The method was to deposit a gold nanoparticle above the fluorine-doped tine oxide surface as a platform to the sensitive COVID-19 antibody by measuring a change in the electrical conductivity. The limit of detection was 90 femtomolar (fM) and 120 fM, sequentially [106].

An electrochemical biosensor for the detection of MERS-CoV coronavirus has been reported. It is focused on a productive test performed with a range of gold nanoparticles adapted to carbon electrodes. An antibody spike protein S1 was used in MERS-CoV as a screening tool. The time of detection was 20 min after sample isolation and purification [107]. A study described the analysis of multiple manufacturing methods, concepts for detection and applications of various biosensors [108]. Electrochemical biosensors have long been used for a wide variety of products in different areas. These biosensors reflect a standard biosensor platform that includes semiconductors and electrodes printed on the screen [109]. These biosensors can be classified into four major groups including potentiometric, amperometric, cyclic and impedimetric to check the changes in dielectron properties, frequency, shape and load distribution [110]. Such biosensors have been used to identify various biological targets such as proteins, cancer biomarkers and nuclear acid [111,112].

Due to its benefits in analyzing a biological sample, the electrochemical sensor has a large potential in the conversion of a biochemical to an electronic signal. However, the isolation and purification of the sample takes time.

#### 4.2.2. Electronic Biosensors

The electron sensor based on field effect transistors (FET) is among several potentiometric methods. It consists of an insulator layer (e.g., SiO_2_) that acts as an independent transducer, which is selective to the target molecule from the biological recognition element. Once the analyte binds to the recognition element, the charge distribution at the surface changes with a corresponding change in the semiconductor’s electrostatic surface potential, which is used to detect nucleic acids and proteins [113].

This section includes one study, a report about the development of an electronic biosensor by using a FET biosensing unit in clinical samples to diagnosis SARS-CoV-2 virus. The sensor was provided by a graphene sheet FET with a unique SARS-CoV-2 spike protein antibody. The sensor output was calculated using an antigen protein cultivated virus and a COVID-19 patient nasopharyngeal swab. The limit of detection was 2.42 × 102 copies/mL in medical tests [35].

Furthermore, these sensors aim to be low-cost and simple/easy to use; however, the coronavirus samples need several pretreatments and filtration processes before the final diagnosis.

#### 4.2.3. Physical Biosensors

This part focus on physical biosensors utilized for the diagnosis of COVID-19. In this section, two articles were collected, which include piezoelectric and magnetic sensors.

A piezoelectric biosensor was improved for the detection of SARS-associated coronavirus (SARS-CoV) by the gas state in sputum. The methods were an antibody SARS-CoV linked above the PZ crystal surface in an established direction through protein. A sample was atomized within an aerosol via an ultrasound. The antibody on the crystal could specially adsorb the SARS antigen and the different mass of crystal would lead to a frequency shift [114].

A new point of care method for particular SARS-CoV-2 antibody detection in blood serum based on magnetic detection has been reported and matched with a test ELISA. The approach was to use columns coated with a SARS-CoV-2 spike protein peptide. The results were a four-fold shorter assay time from the test ELISA. The time duration to diagnosis was 42 min [115]. A review study of popular biosensor systems based on magnetoresistance, magnetic particle spectroscopy sensors and nuclear magnetic resonance have been reviewed to prevent the outbreak of the SARS-CoV-2 virus [116].

However, this method’s challenges are a lack of accuracy, sensitivity or quantitative measurement probability; sample isolation and purification are time-consuming. The sensing system required substantial isolation of equipment due to its high environmental sensitivity, minimizing obstacles such as vibration. These biosensors have been used to detect targets including hormones, bacteria and cells in a wide variety of applications [117].

#### 4.2.4. Optical Biosensors

This category deals with articles on optical fiber sensor methods and applications to detect the COVID-19 virus. A significant number of optical biosensors are based mainly on the surface plasmonic resonance concept [118] including when optical components such as waveguides are used in modulation principles [119], based on a photon crystal fiber (PCF) [24], fiber optics based on wavelength [120] and by using nanolaser [23] and are categorized into optical sensors. Typically, the optical sensor consists of a light, detector and optoelectronic transducer.

According to studies published, optical biosensors such as surface plasmon (SPR) as well as LSPR have been available on the market since the early 1990s and have been used extensively in detecting viral strains such as those correlated with SARS and MERS in lab conditions [121,122].

In developing an optical sensor LSPR for viral RNA samples, the researchers created an alternate research approach utilizing a biosensor. For stability, the sensor mixed two different effects: optical and thermal. Based on nanoparticle gold constructs on a glass substratum, the biosensor artificially created DNA receptor sequences complementing RNA genome parts of the SARS-CoV-2 virus. These unique sequences were grafted onto the gold nanoislands, detecting SARS-CoV-2 reliably. The team warned, however, that further improvement was required before application [45].

A study to develop an optical fiber sensor based on evanescent wave absorbance (EWA) for fast and specific detection of COVID-19 to purpose a point of care was shown. The approach was based on two suggestions. The first included a measure of host immune reply and the second, the diagnosis of viral cell surface proteins utilizing fitting receptors. However, the host immune response was not accurate indicators of the current COVID-19 virus and other respiratory pathogens such as SARS-CoV and MERS might trigger similar reactions [39].

A platform of a fiber optic absorbance biosensor (P-FAB) was proposed to diagnose COVID-19 in the saliva sample direct with minimum pre-processing. The approach was based on the changing intensity/absorb of light reflected inside the fiber optic probe on a U shape. It used a green light emitting diode because AuNP absorb reaches between 520–545 nm. However, some limitations included enhancing a few parameters and the duration time to detect within 15 min at a small concentration sample [41].

An improved optical biosensor for localized surface plasmon coupled fluorescence (LSPCF) for SARS-CoV detection was reported. The detection limit was 104-fold at a low concentration (∼1 pg/mL) of COVID-19 (N) protein in serum through three hours. However, the need for isolation and purification of the sample takes time [121]. Using a SARS-CoV detection quantum dot-conjugated biosensor test chip, the duration time for diagnosis was 1 h after sample separation and washing [123].

A simple, reduced price, sensitive molybdenum disulfide (MoS2) biosensor has been developed. The biosensor was based on a fluorescent immunosensor, which used the coronavirus detector for fluorescence energy resonance transfer (FRET) and showed 4.6/10^2^ per mL sensitivity [124].

##### Analysis Outcome of the Diagnosis of COVID-19 Based on Biosensor Application

An analysis of 12 studies showed an in-depth study of comprehensive types of biosensor technology for coronavirus detection. It included related articles as shown in Table 3. It is important to establish a sensitive and specific analytical system that monitors against the spread of COVID-19 in the environment. Therefore, optical biosensors with all of the above benefits will play a significant role in environmental research. One of the difficulties of utilizing optical fibers to detect disease is that temperature fluctuations will affect the material’s optical properties. Thus, sensors built are mostly to operate at specific temperatures and provide incorrect readings if the sample becomes hotter or colder. Temperature insensitivity allows the sensor to be more suitable for outdoor applications.

Consequently, the option of laser spectroscopy for sensing must be based on many parameters considering critical hypotheses such as the sample type, for example, solid, liquid, gas, powder, aerosol, a mixture and spectroscopy category (molecular or atomic).

The outbreak of coronavirus disease COVID-19 indulges challenges on the continuance of activities globally. This pandemic affects many people and continuously creates health problems. However, there are successful biosensors but the development of reliably is needed. A laser with artificial intelligence for virus detection is more potent than conventional techniques such as chromatographic techniques for the environmental monitoring of pollutants. That could potentially track and detect the virus causing infectious diseases at an early stage of the infection. However, the full potential of biosensors and characterization methods are yet to be explored for on-site usage, given their widespread usability and well-known academic benefits. Scientific obstacles and opportunities for biological/chemical sensors definitely remain before they meet the demands of reliable, accurate and early detection in infectious diseases. Virus detection can be divided into four major categories:

- Direct virus identification. A perfect virus is detected only by biosensors or more generally by cultured cell techniques [125].
- Viral recognition of RNA/DNA using RT-PCR and PCR principles, whether attributed with fluorescence in regular nuclear acid platforms or using advanced methods including LSPR, SPR, QCM and other sensor techniques [45].
- Detection of an antibody or antigen. Bioassays use absorption coefficient monitors and many optical and electronic biomedical sensors that basically calculate molecular kinetics. High-resolution scanning probe microscopy with a 1000-fold resolution less than the optical wavelength range in the size of a fraction of a nanometer is used for the surface properties of viruses [126].
- Tools to enhance surface characterization electromagnetic techniques. The virus surface is imaged with a focused electron beam to identify topographic characteristics [127]. X-ray crystallography (XRC) virus features are identified to determine 3D virus structures [128].

## 5. Enhancement Design of Sensor Output Performance

In this section, the essential parameters to improve biosensor output performance when producing is described. However, these parameters depend on the analysis method, the configuration set-up, the elements deposited as thin films and even how good the instrumentation is. In addition, most of the studies published on optical biosensors are presented such as the sensitivity to the surrounding refractive index (SRI), signal noise ratio, spectral bandwidth and the figure of merit (FOM). To compare the adjustable in evaluating these parameters in assessing the performance of optical detection, we will examine their association with the limit of detection (LOD).

### 5.1. Sensitivity

The sensitivity of the biosensor by ratio shifting wavelength for the transmission of light or attenuation in changes surrounding the refractive index is defined. The shifting of wavelength in incident light occurs due to the change of the refractive index and the thickness of the waveguide coating layer. The adhesion of a thin film nanoparticle deposited above the bioreceptor layer is used to improve the sensitivity of the optical sensor, thus this parameterization will also depend on the refractive index of the material chosen as expressed in Equation (1) [129].

(1) S=Δλ/Δn.

### 5.2. Signal Noise Ratio

Spectroscopic data are selected either through defining the deflection height in the time domain or defining the amplitude in the frequency-domain received signal. Thus, chemical graphs are obtained via illuminating the sample at a constant wavelength and plotting the movement as a function. Typically, many pulses are equalized at any point to increase the signal noise ratio (SNR). Methods for producing a good SNR are of considerable importance because they can improve the usually low efficiency of scanning tests by reducing the gain time due to photothermal induced resonance (PTIR) signal changes in both time and frequency. However, noise can be lowered through implementing a time-frequency signal transformation such as the Morlet wavelet transform and filtrate the signal as times longer. According to the hypothesis [130,131], the PTIR signal (S), is equivalent to the absorbed energy through a unit area ( $U_{abs}$ ), the cube of the sample thickness ( Z ) and the sample thermal expansion coefficient $\left(\alpha_{exp}\right)$ . This is inversely proportional to the sample thermal conductivity ( η ), as shown in Equation (2):

(2) $$S\sim \frac{\alpha_{exp}}{\eta }\cdot U_{abs}\cdot Z^{3}.$$

A unit area depends on the evanescent field intensity inside the sample, where (*E*) is the amplitude field in the sample, typically expressed as a function of the penetration depth ( $d_{p}$ ). The total internal reflection is shown in Equation (3).

(3) E=E0·e−Zdp

where (*E*_0_) is the electric field amplitude and $d_{p}$ is the distance where E is reduced by a factor of ( e ) as shown in Equation (4).

(4) dp=λ2·π[(n12·sin2θ−n22+k22)2+(2·n2·k2)2+(n12·sin2θ−n22+k22)2]−12

where ( θ ) is the light incident angle, $n_{1}$ and $n_{2}$ , are the refractive index of the Attenuated total reflection (ATR) between element and the sample, respectively. $k_{2}$ is the extinction coefficient of the sample. The thermal conductivity can be shown as the concentration-weighted middle of the element thermal conductivities ( $\eta^{A}$ , $\eta^{B}$ ), as shown in Equation (5).

(5) $$\eta^{max}=C_{A}\cdot \eta^{A}+\left(1-C_{A}\right)\cdot \eta^{B}.$$

### 5.3. Full Width at Half Maximum/Minimum (FWHM) and Q-Factor

The center wavelength of a transmission is the location of the maximum or minimum value of that band and is also called the resonance wavelength ( $\lambda_{0}$ ). The FWHM is defined at a level 3 dB up/below, depending on whether the measurements are in transmission or absorption. The quality factor ( Q -factor) is a parameter closely linked to the FWHM, which is essentially the result between $\lambda_{0}$ and the FWHM [131,132] as shown in Equation (6).

(6) $$Q=\frac{\lambda_{0}}{FWHM}.$$

Accordingly, the Q -factor is a reliable indicator of the resolution of the analyses together with the resolution of the detector.

### 5.4. Figure of Merit (FOM)

After defining the sensitivity and FWHM, the function of thickness is defined. This rate is an idea of how much the sensor is decreasing the perfect position. The figure of merit ( FOM ) can be measured as the result between these two, as indicated in the expression in Equation (7).

(7) $$FOM=\frac{S}{FWHM}.$$

### 5.5. Detection Limitation

The limit of detection (LOD) is the smallest volume of target microorganisms or genome copies that can be probably detected under optimal conditions and is a necessary step in defining the sensitivity of any test. Detection limitation is considered one of the main criteria analyzed with the design and description of optical biosensors. It shows the hypothetical minimum rate of the analyte concentration that the sensor can detect under perfect laboratory conditions. Here, in framework sensitivity, we will discuss the point of identifying the detection limit and will include details on how to evaluate this criterion when evaluating the experimental design [133].

It is necessary to decide how well the experiment can detect lower concentrations of the target strain, especially if the strain has a low infectious level during the production of a test or diagnostic tool used to determine the presence of a specific pathogen. The techniques used to install the LOD can improve depending on test type and use. Measuring the clinical or environmental detection limit is usually linked with several challenges including the potential for ecological inhibitors, loss of the organism or the presence of impurities. At each step of the recovery process, there is the potential for sample loss, which directly affects the LOD. However, it can measure the signal at the LOD, as expressed in Equation (8).

(8) $$y_{LOD}={\bar{y}}_{blank}+t_{a,k-1}\;s_{y}$$

where $t_{a,k-1}$ is the α-quantile of t-Student function with k−1 degrees of freedom where (1 − α) defines the confidence interval, k at this concentration. The American Chemical Society suggests k = 10 and $s_{y}$ the mean value and the standard deviation that is correlated. A second adapted solution, which is a fusion of the two existing methods, is recommended. The LOD can be extracted from the curve of adjustment through the inverse of the function $f^{-1}$ and can be obtained according to this procedure as shown in Equation (9).

(9) $$y_{LOD}=f^{-1}\;\left({\bar{y}}_{blank}+3\sigma_{max}\right).$$

Finally, the third strategy for calculating the LOD form is shown Equation (10).

(10) yLOD=RSsurf=RΔnρmax

where R is the sensor resolution and ( $S_{surf}$ ) is the result between the RI change (Δn) and the highest surface mass concentration ( $\sigma_{max}$ ) of the aim analyte. This the third approach is popular for SPR-based biosensors.

## 6. Monitoring and Diagnosing the COVID-19 Virus in the Environment

The previous three alarming clusters of early 21st century new human coronavirus infections have demonstrated the value of easily accessible, reliable and rapid testing technology to control emerging and re-emerging pandemics. The mixture of other particles in the air complicates the detection of the existence of infectious virus particles. The atmosphere comprises of a significant number of circulating particles, most of which are organic. Usually, you inhale about a thousand biological particles with each intake.

There are many viruses in the environment such as bacteria, fungi, pollen and animal and plant debris. The smallest of all particles are viruses. They range in size from 10 to 300 nm. In contrast, red blood cells average about 6–8 microns, bacteria range from 1–4 microns and fungi range from 5–10 microns.

The laser-based optical detection imaging considers the best solution to detect COVID-19 viruses on the surface and environmental protection; thus, many studies have proved developments of lasers to detect viruses that can be persistent and accurately monitor healthcare. A sensor that combines laser line illumination efficacy with fluidic confinement advantages can be used to track nano-objects [134]. The following showed a study of laser spectroscopy techniques with advanced femtosecond methods for a single viral detection that were all too apparent [135]. Individually trapped viral particles were studied. Double nanohole (DNH) apertures in a gold film have been used to trap one of the smallest reported virus particles, which was 25 nm in diameter [136]. The replacement of fluorescent quantified antibody-based probes with laser detection probes would create a new platform for quantifying biomarkers based on optical instead of enzyme amplification. Virus laser bridges synthetic biology and laser physics. Probes display 10,000 times more signal from only a 50% increase in probe concentration [137]. Surface-enhanced Raman scattering (SERS) technology was applied to flu virus detection. SERS has a 10^6^–10^9^ times signal amplification, which provides excellent sensitivity for precise influenza virus identification [138]. Reliable viral detection, sizing and filtering is essential for biosensors, environmental monitoring and quality control.

Optical biosensor technology is stable, flexible and sensitive. In a pandemic outbreak, it may be used to store our health care system critical data that can be utilized with any related pandemic such as COVID-19. It can be used effectively as a multi-sensor, smart network to control remote monitoring of COVID-19 propagation. This biosensor patch can be used to diagnose early and track COVID-19 in the built environment. This patch can conduct real-time temperature tracking, ECG traces, respiration rate, etc.

**Figure 8:**
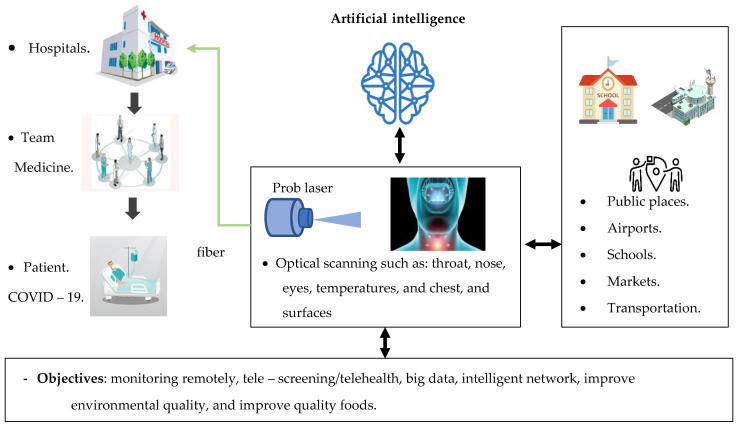
Proposal framework for monitoring and the detection of coronavirus disease (COVID-19) for environmental and telehealth applications.

However, 3D scanning laser transforms physical elements into digital computer data. This technology was successful in reverse engineering systems. In medicine, this technology is used to scan the human body and its part in the exact dimension. A 3D scanning output is used to examine real-world structures and capture form and appearance details. The 3D model optical scan can be built with AI and employed to chest screening for COVID-19 as it is a non-contact procedure. It is also a valuable method to diagnose and measure COVID-19 in the environment to assist medical teams remotely. This data can be used for multiple purposes often helpful in designing 3D scanners in other applications such as thoracic digital reality, motion control, autonomous imaging and industrial design. Our proposal framework for monitoring and detecting coronavirus disease (COVID-19) can assist medical teams by providing remote monitoring and fast detection in real-time, improve the quality and accuracy of optical biosensors, improve the quality of health care in public places such as schools, markets and airports and improve the quality foods, as shown in Figure 8.

## 7. Challenges of the Techniques for Detecting COVID-19

This section discusses the limitation of many detection techniques associated with COVID-19 virus testing and validation, as shown in Table 4. The literature on detecting coronavirus disease based on image techniques and biosensor applications has evolved over the last five years. Researchers in this field have addressed many problems; for example, real-time transcriptase polymerase chain reaction (RT-PCR) dependent tests on lung samples are the gold standard for COVID-19 diagnostics. However, the challenges of detection by a molecular model, taking longer with a variety of filters and insulation stages to extract viral RNA, are performed for the fluid collected. In addition, it damages sample RNA through opening its viral capsid. One factor could be the host’s immune response. That leads to the introduction of tiny RNA fragments into the blood, which is difficult for RT-PCR identification [139,140,141].

### 7.1. Collection and Transport of Samples

Specimens are closely connected to detection performance and precision. The upper respiratory tract sample methods include a swab of the throat and lower respiratory tract include a nasal swab, deep throat saliva and sputum.

The important issues of diagnosis are sample collection and transportation from a patient related to the sample collection are individually important and deserve focus. Although the nasopharyngeal or throat swab is diagnosable, sampling can make the patient disturbed and cause aerosol inducing coughing and sneezing, presenting a possible health hazard for healthcare workers. In addition, misidentification such as sample pollution, manual (e.g., swab) errors, collection of inadequate material for quality or volume, treatment, transport and storage, lack of samples and the presence of interfering substances may be important causes of diagnostic errors [142]. Another research paper shows a slightly higher favorable performance of sputum swabs (76.9%) than swabs on the throat [143].

### 7.2. AI Image Techniques

Artificial Intelligence (AI) is an important weapon that can be extremely helpful toward COVID-19 pandemics on population risk management and screening. It is an algorithm close to machine learning, computer vision and natural language processing that can teach machines to use large data models to detect, describe and forecast trends. Today, the usage of this technology is limited as there is a shortage of evidence. Perhaps the data are really loud and obsolete. A lack of data hampers the use of AI for a diagnosis of COVID-19 [144]. The computer tomography used for a CT scan and X-ray are the most famous to diagnose the SARS-CoV-2 virus; however, the visibility of the scan decreases as the infection spreads, with cases that are often reported as abnormal patterns in the scan [145,146].

AI can forecast an epidemic, minimizing or even halting the virus transmission. False details on COVID-19-related social media can be identified and ultimately deleted with AI applications. Using AI can optimize clinical trials for medications and vaccinations against this strain. It can build robotics that can help perform sanitization jobs and online medical reviews of people. This technology will generate CT scans or X-rays needed to diagnose viral pneumonia. Using this technology is useful to produce the devices required for the healthcare system.

### 7.3. Biosensors

Biosensors have been used to transform biological signals into electrical signals. In our review article, many important biosensor forms are included in diagnosing COVID-19 such as optical, thermal, piezoelectric and electrochemical biosensors. These are found in a wide range of areas such as medical research, environmental and communication.

In the current COVID-19 pandemic, biosensors can provide instruments that can be simple to use, adaptive, cost-saving and have high precision. A glucose detector is a fine illustration of a biosensor used in clinical study and disease detection. However, there is some limitation in using biosensors to detect COVID-19 as it does not solve it yet and is time-consuming because of taking samples from patients and needing isolation, washing and filtration to provide for detection. The biochemical technique is used to detect viruses by protein-protein interactions. One major problem, however, is that while the amount of viral load varies throughout infection, it may be difficult to detect low concentrations of viral protein [147].

Optical biosensors are categorized mainly as optical sensors based on the concept of the plasmon such as the SPR as well as the LSPR. Advanced surface chemistry methods developed with plasmon detect virus strains, provide excellent accuracy and rapid response times. However, they remain difficult to use in care point applications. Diagnostic methods most for viral infections use DNA, RNA and antibodies or antigens, which are considered as the gold standard for viral diagnosis. In the state of pandemics such as the COVID-19 virus, this process has the problem of being time-consuming. Therefore, when a fast diagnosis is required, the tissue culture procedure seldom is considered. However, when study is needed, this approach is the best for detecting viruses and for isolating from cells and examining the response of any host to viral infection [148]. This review paper can be a good starting point for researchers to understand the detection of coronaviruses disease (COVID-19) limitation and control outbreak.

## 8. Conclusions and Future Perspectives

The worldwide pandemic of COVID-19 majorly affects life. There has been a noteworthy increment in the number of infections in individuals worldwide. Several nations, government and researchers are attempting to adapt to this worldwide emergency. This review summarizes a comprehension of infection transmission of SARS-CoV-2 and knowledge of the state of art diagnostic methods approach based on biosensor applications, artificial imaging techniques and mentioned challenges with a viewpoint. It can be concluded that the diagnosis of a coronavirus location on surfaces by joining an optical sensor with a picture innovation speaks as the best answer for decreasing the pandemic. However, the sheer size of information growing every day about COVID-19 is so bountiful and dynamic that even clinical and medical teams and media cannot stay off this new pandemic. As can be seen, connection between hospitals and different locations by using a smart optical network to collect the big data from probe lasers is distributed at point ends. Finally, in future perspectives, potential laser-based optical detection imaging helps environment monitoring, analysis of virus concentration in the air and food quality testing. Thus, data can be collected by an intelligent network of medical biosensors for COVID-19 tracking to improve healthcare quality.

## Figures and Tables

**Table 1:** Analysis of category styles of transmission coronavirus.

Transmission Style	Hazard	Places	Data Published	Country	Finding	Reference
Environment to human	High	Plastic, stainless steel, copper and cardboard	17 March 2020	USA	SARS-CoV-2	[51,56]
Environment to human	High	Hospital rooms	29 May 2020	Singapore	SARS-CoV-2	[52,58]
Environment to human	High	Air and surface	26 June 2020	Italy	SARS-CoV-2	[53]
Environment to human	High	Surface contamination	2016	London	SARS-CoV and MERS-CoV	[54]
Environment to human	High	Air, surface	7 May 2020	China	COVID-19	[55]
Environment to human	High	Water	28 April 2020	Italy	SARS-CoV-2	[57]
Environment to human	High	Toilet	13 August 2020	China	SARS-CoV-2	[59]
Human exchange	High	Contacts	24 January 2020	China	SARS-CoV-2	[61]
Human exchange	High	Ocular surface	22 April 2020	Australia	SARS-CoV-2	[63]
Human exchange	High	Dental clinics	19 February 2020	China	SARS-CoV-2	[66]
Animal to human	Medium	Bats	2020	USA	SARS-CoV-2	[73,74]
Animal to human	Medium	Sold avian, swine, porcine, bovine, canine, seafood, frogs, camels	2020	China	SARS-CoV-2	[71]
Human to others	Low	Wastewater surveillance	18 April 2020	Australia	SARS-CoV-2	[76,78]

**Table 2:** Analysis of the diagnosis of COVID-19 based on artificial intelligence techniques.

Type of Dataset		AI Techniques		Case Study	Efficiency of Detection (%)	Installation Data	Collection Dataset Size	AI Partition			No. of Classes
Prime Data	Minor Data	Traditional Machine Learning Techniques	Deep Learning Techniques								
								Test	Validation	Training	
✕	✕	✕	✓	CT scan	96%	19 February 2020	2 images	✕	✕	13.3%	COVID-19, Flu
✓	✕	✓	✕	CT scan	93.6%	2020	13,500 images	✕	✕	✕	COVID-19
✓	✓	✓	✓	CT scan	50%	2020	NA	✓	✕	✕	COVID-19
✓	✓	✓	✓	CT scan	NA	20 February 2020	NA	✕	✕	✕	COVID-19
✕	✓	✕	✓	X-ray	87.02%, 98.08%	26 April 2020	127 images	✕	5-fold cross	✕	COVID-19, no finding and pneumonia
✕	✓	✕	✓	X-ray	98.03%	21 April 2020	2839 images	10%	10%	80%	COVID-19, normal, pneumonia
✕	✓	✕	✓	X-ray	86.9%	16 June 2020	430 images	0.7	0.1	0.2	Normal, bacterial tuberculosis, viral and COVID-19
✕	✓	✓	✕	X-ray	80%	2020	8 images	✕	two-fold	✕	COVID-19
✕	✓	✓	✓	X-ray	99.27%	2 May 2020	845 images	30%	5-fold cross	70%	coronavirus, pneumonia and normal
✓	✓	✓	✓	X-ray	87%, 98%	2020	1144 images	30%	✕	70%	Normal, COVID-19, MERS SARS, Varicella, Streptococcus Pneumocystis

**Table 3:** Deep analysis for the detection of COVID-19 based biosensors.

Sensor Type	Application Range	Material (nm)	Prognosis	Diagnosis	Installation Date	Target	Duration	Detection Limit	Sample Size	Detection of COVID-19 Virus	
										In Clinical	On the Surface
Optical LSPR PPT	SARS-CoV-2	Gold	✓	✓	8 Apr 220	DNA	800 s	0.22 pM	0.01 pM–50 Μm	✓	✕
FET sensor	SARS-CoV-2	Graphene	✓	✓	29 May 2020	Spike protein	4 h	1.6 × 101 pfu/mL	1 fg/mL	✓	✕
Magnetic	SARS-CoV	NA	✓	✓	3 July 2020	Anti-spike protein	42 min	2.96 ng/mL	2 ng mL^−1^	✓	✕
P-FABU-bent optical fiber	COVID-19	Gold	✓	✓	1 June 2020	N-protein	15 min	10–18 M	106 particles/mL	✓	✕
Electrochemical	SARS-CoV-2	Gold	✓	✓	11 M ay 2020	Spike protein	10–30 s	90 fM	1 μM	✓	✕
Electrochemical	MERS-CoV	Gold	✓	✓	27 Feb 2019	Spike protein	20 min	1.0 pg mL^−1^	10 μg mL^−1^	✓	✕
Optical Fiber	COVID-19	Gold	✕	✓	11 June 2020	IgG-type antibodies	1 h	100 units/mL	NA	✓	✕
Piezoelectric	SARS-CoV	Crystal with quartz wafer	✕	✓	2004	Antigen (sputum)	1 h	0.6 µg/mL	0.6–4 µg/mL	✓	✕
Optical LSPCF	SARS-COV	Gold	✓	✓	2009	Nucleocapsid protein	3 h	1 pg/mL	∼1 pg/mL	✓	✕
Optical	SARS-CoV	Quantum dots	✓	✓	24 July 2011	Nucleocapsid protein	1 h	0.1 pg/mL	0.1 pg mL^−1^	✓	✕
Nanocrystals optical	Coronavirus	Zirconium quantum dots	✕	✓	29 Oct 2018	Antibodies	1 h	79.15 EID/50 mL	1000 EID/50 mL	✓	✕
LFA	SARS-CoV-2	Gold	✕	✓	21 May 2020	IgM antibody	15 min	NA	10−20 μL	✓	✕

**Table 4:** Categories of challenges for the detection of COVID-19 techniques.

Challenges, Detection of COVID-19 Virus Techniques According to Critical Review Research		
Virus Detection Categories	Techniques	Limitation
Indirect detection: DNA, RNA	Electronic sensors	Signal transduction process found is not always apparent. Heterogeneous interface structures. Long time result.
Indirect detection: DNA, RNA	Magnetic sensors	These require several washing steps, and a well-trained technician is necessary. Sensitivity medium and time-consuming.
Indirect detection: Spike protein	Electrochemical sensors	Immobilization method of the concerned nanomaterial to minimize the chance of error. Needs a long time.
Indirect detection: IgM antibody, DNA, RNA	Optical sensors: LSPR, P-FAB, EWA, QCM, SPR and LSPCF	Requirement for point of care remains difficult. High cost.
Direct detection: CT image	CT scan	The opacity of ground-glasses. Irregular linear patterns in the scan. Lacking in a sample dataset.
Direct detection: X-ray image	CXR	Abnormalities of the radiograph. Foggy opacity. A sensitivity of 59%. Lacking in a sample dataset.
**All Detection Techniques of COVID-19 Viruses Based On**		
**Viral RNA, DNA Identification**	Long time result; can take 4 h to 3 days. Errors in sampling. Sample preparation, isolation, washing and analysis.	
**Antibody or Antigen Identification**	Low concentration and homogeneous protein. Lacking sensitivity.	

## Abbreviations

**Abbreviation**	**Description**
COVID-19	Coronavirus disease
MERS-CoV	Middle East Respiratory Syndrome
SARS-CoV-2	Coronavirus Severe Acute Respiratory Syndrome 2
SARS-CoV	Coronavirus Severe Acute Respiratory Syndrome
2019-nCoV	2019 novel coronavirus
RT-PCR	Reverse Transcription Polymerase Chain Reaction
LFR	Low-Frequency Raman
UFOP	U-bent Fiber Optic Probe
P-FAB	Plasmonic Fiber-Optic Absorbance Biosensor
FET	Field Effect Transistor
Zr QDs	Chiral Zirconium Quantum Dots
PPM	Parts Per Million
QCM	Quartz Crystal Microbalance
LFA	Lateral Flow Assays
